# Wogonin, a Bioactive Ingredient from Huangqi Guizhi Formula, Alleviates Discogenic Low Back Pain via Suppressing the Overexpressed NGF in Intervertebral Discs

**DOI:** 10.1155/2023/4436587

**Published:** 2023-02-20

**Authors:** Jitian Li, Weifeng Duan, Shuang Chai, Yage Luo, Yan Ma, Ning Yang, Man Liu, Wei He

**Affiliations:** ^1^Guangzhou University of Chinese Medicine, Guangzhou, Guangdong 510006, China; ^2^Henan Luoyang Orthopedic Hospital (Henan Provincial Orthopedic Hospital), Zhengzhou, Henan 450001, China; ^3^Henan University of Chinese Medicine, Zhengzhou, Henan 450001, China

## Abstract

**Purpose:**

To investigate whether wogonin, a key bioactive ingredient of Huangqi Guizhi formula (HQGZ formula; a Traditional Chinese Medicine herbal formula) according to network pharmacology analysis, has analgesic effects on discogenic low back pain (LBP) via regulating the nerve growth factor (NGF) in intervertebral discs (IVDs).

**Methods:**

The lumbar IVDs of rats were punctured to discogenic LBP, and the therapeutic effect of orally administrated HQGZ for discogenic LBP was investigated by measuring mechanical and cold allodynia and histological analysis. A network pharmacology analysis was conducted to search for bioactive ingredients from the HQGZ formula, and wogonin was suggested to be the most possible bioactive ingredient for LBP treatment. Subsequently, the analgesic effect of wogonin was investigated in the LBP model, and the gene expression of propain peptides in the bilateral dorsal root ganglia was analyzed using RT-PCR. Finally, immunohistochemical staining was performed for NGF expression of NGF in the IVDs to determine whether wogonin treatment would ameliorate NGF-induced LBP.

**Results:**

Oral administration of HQGZ for two weeks significantly ameliorated puncture-induced IVD degeneration (IDD) and LBP. In addition, the network pharmacology analysis revealed that wogonin, quercetin, and kaempferol were the potential candidate components of HQGZ for LBP treatment. Furthermore, we proved that wogonin had significant analgesic effects in the LBP model. Finally, wogonin was demonstrated to suppress the upregulated NGF in the IVD and ameliorate NGF-induced LBP in rats.

**Conclusions:**

The HQGZ formula has significant analgesic effects for LBP. In addition, the bioactive ingredient of wogonin was extracted from HQGZ and ameliorated LBP by suppressing the overexpressed NGF in degenerated IVDs. Therefore, wogonin has potential to be alternative treatment for LBP in clinical.

## 1. Introduction

Low back pain (LBP) affects billions of people all over the world and results in a large disease burden [1]. An epidemiological study estimated that the one-year incidence of a first-ever episode of LBP was 6.3–15.4%, while the estimated 1-year incidence of any episode of LBP was 1.5–36% [2]. However, effective prevention and treatment methods for LBP are inadequate, and search of effective new drug or bioactive ingredient will be a significance for the treatment of LBP.

In addition to anti-inflammatory drugs and opioids for LBP [3], several Chinese traditional herbal recipes have significant therapeutic effects for intervertebral disc (IVD) degeneration and LBP [4]. Our institute developed a unique recipe called Huangqi Guizhi (HQGZ) formula to effectively treat acute or chronic LBP based on traditional Chinese medicine (TCM) theory. The HQGZ formula mainly includes the herbs *Scutellaria baicalensis*, *Ramulus Cinnamomi*, and *Achyranthes bidentata*. Although there is adequate clinical experience with its use, more animal data and evidence regarding its effects on the pathophysiological mechanism are needed to prove the effects of HQGZ on LBP.

Many chemical components are extracted from the herbs of the HQGZ formula. Therefore, we conducted a network pharmacological analysis to target the most likely potential candidate. Our results suggested that wogonin may be the primary bioactive ingredient in the formula for LBP treatment. Wogonin is a natural flavone extracted from the root extract of *Scutellaria baicalensis* [5] and *Achyranthes bidentate* [6]. It was traditionally applied to treat inflammatory-related diseases [7]. For example, it was suggested that wogonin can inhibit carrageenan-induced inflammatory activity or inflammation of dorsal root ganglion (DRG) neurons with lipopolysaccharide treatment [8, 9].

One of the potential mechanisms for LBP involves upregulation of pain-related peptide, for example, nerve growth factor (NGF) [10, 11]. For example, NGF participated in bacteria-induced or degeneration-related LBP [10, 11]. Thus, we hypothesized that wogonin may attenuate LBP via regulating the NGF expression. This study was the first time to investigate the therapeutic effect of wogonin for LBP and would give more choice for LBP treatment.

## 2. Materials and Methods

### 2.1. Network Pharmacology Analysis

To identify compounds in each herb of the HQGZ formula, we search the Traditional Chinese Medicine Systems Pharmacology Database and Analysis Platform (TCMSP, https://tcmsp-e.com/tcmsp.ph, V2.3). We used the index of oral bioavailability and drug likeness to search the active ingredients in the TCMSP database. In this study, we fixed the oral bioavailability ≥ 30% and drug likeness ≥ 0.18 as the criteria to identify the bioactive ingredients and targets. Then, “Low back pain” was used as a search term to identify the relevant targets of LBP from the GeneCards database (https://www.genecards.org/), DisGeNET database (https://www.disgenet.org/), OMIM database (https://omim.org/), and PharmGKB database (https://www.pharmgkb.org/).

The bioactive ingredients and target of the HQGZ formula and LBP targets were imported into the online drawing website (http://bioinformatics.psb.ugent.be/webtools/Venn/) for visualization. The intersection was considered to indicate the potential targets of HQGZ formula for LBP treatment. Then, the bioactive ingredients and the potential target of the HQGZ formula were analyzed with Cytoscape 3.8.0 software to draw a bioactive component-target-network and identify the potential factor with a key role in HQGZ treatment.

In addition, PPI (protein-protein interaction), GO (Gene Ontology), and KEGG (Kyoto Encyclopedia of Genes and Genomes) enrichment analyses were analyzed with an online gene annotation and analysis resource Metascape (https://metascape.org/gp/index.html#/main/step1) to demonstrate the underlying molecular mechanisms. A threshold of *p* < 0.05 was set for the key GO and KEGG pathways.

### 2.2. Establishment of an LBP Model in Rats

Before the animal experiment, we got the authorization from the Institute of the Animal Care and Use Committee of Henan Luoyang Orthopedic Hospital and strictly complied the National Institutes of Health guidelines for the care and use of laboratory animals (NIH Publications No. 8023, revised 1978). Based on our previous protocol [12], male rats weighing 250–300 g were used for the animal study. To induce discogenic LBP, the lumbar intervertebral discs at the level of 4-5 were punctured using an 18 G needle. Meanwhile, the caudal IVDs at the level of 3-4 were punctured for histological analysis using the same procedure. Five groups were assigned as follows: the sham-surgery group, the puncture group, the puncture+HQGZ group, the puncture+wogonin group, and the puncture+GW441756 group. Meantime, a 28-gauge microsyringe was used for NGF injection to avoid physical discogenic LBP and anatomical damage to IVDs, and the rats were divided into the sham-surgery group, NGF group, and wogonin+NGF group.

### 2.3. Drug Administration

In humans, HQGZ formula (approved ID: 991360; Henan Luoyang Orthopedic Hospital, Henan, China) is orally administrated with a dosage of 300 mg/kg (for patients with a body weight of 60 kg, 1.8 g of HQGZ was given daily). Thus, in rats, HQGZ was intragastrically given with a dosage of 300 mg/kg/day (HQGZ was dissolved into 1 mg/ml). Wogonin (cat. no. 632-85-9; Chengdu Must Bio Technology Co. Ltd., Chengdu, China) was dissolved with 0.5% CMC-Na (the sodium carboxymethyl cellulose with viscosity at 800–1200 mPa.s; cat. no. HY-Y0703; MedChemExpress Inc., NJ, USA) into 10 mg/ml. The rats received wogonin at a dose of 50 mg/kg/day via an intraperitoneal injection. In addition, GW441756 (cat. no. 504433-23-2; Selleck Chemicals, TX, USA) was given with a dosage of 20 *μ*g per disc, and NGF (cat. no. 556-NG; R&D Systems, MN, USA) was applied with a dosage of 3 *μ*g per disc using X-ray-guided percutaneous injection into IVDs. An illustration of timeline and workflow was presented in the figures.

### 2.4. Measurement of Paw Withdrawal Threshold for LBP Assessment

To assess the severity of LBP in rats, the test of paw withdrawal threshold was conducted to calculate mechanical and cold allodynia, which as a credible index for LBP [13]. In brief, there was 10 min for rats to adapt to the surroundings and keep calm before the test. Then, specific filaments (calibrated von Frey filament, Stoelting, Wood Dale, IL, USA) were used to cause sufficient mechanical force on the surface of rodent hind limb following our previous protocol. If the rats bite or lip the paw after stimulated with a specific diameter filament, it was defined as positive reaction (also named as brisk movement). A smaller diameter filament was used when the rats had the positive reaction; otherwise, a larger diameter filament was used. Following the method proposed by Chaplan et al., the mechanical withdraw threshold was calculated and recorded. To conduct cold allodynia, the evaporation of acetone with 0.05 ml around the plantar surface of the hind paw would cause positive reaction, and the percentage of positive reaction was calculated. Assessment was conducted at 3, 7, 11, and 14 days after surgery.

### 2.5. Real-Time Quantitative PCR

In accordance with our previous study [10], rat L1 to L3 DRGs were harvested to analyze the gene expression of substance P (SP) and calcitonin gene-related peptide (CGRP), because bilateral L1–L3 DRGs innervate the lower IVDs [14]. In brief, the TRIzol reagent (Invitrogen, Life Technologies Corporation, CA, USA) was used to extract the total RNA, and the cDNA was produced with reverse transcriptase kit (TaKaRa, Shiga, Japan). The next qRT-PCR was conducted using SYBR Premix Ex Taq Kit (TaKaRa, Shiga, Japan) in the ABI 7500 Sequencing Detection System (Applied Biosystems, CA, USA). In this process, the reaction condition was set as follows: 40 cycles of denaturation at 95°C for 5 s and amplification at 60°C for 24 s. In this study, the primer sequences were designed and used: rat *GAPDH*: forward 5′-ATGACTCTACCCACGGCAAG-3′ and reverse 5′-TACTCAGCACCAGCATCACC-3′; rat *SP*: forward 5′-TGGTCAGATCTCTCACAAAGG-3′ and reverse 5′-TGCATTGCGCTTCTTTCATA-3′; rat *CGRP*: forward 5′-TCTAGTGTCACTGCCCAGAAGAGA-3′ and reverse 5′-GGCACAAAGTTGTCCTTCACCACA-3′; human *GAPDH*: forward 5′-CAGGAGGCATTGCTGATGAT-3′ and reverse 5′-GAAGGCTGGGGCTCATTT-3′; and human *NGF*: forward 5′-GCAAGCGGTCATCATCCCATCC-3′ and reverse 5′-TCTGTGGCGGTGGTCTTATCCC-3′.

All reactions had triplicate repeat and the housekeeping gene was GAPDH. To normalize the targeted gene expression, the 2^-△△Ct^ method was used when compared with the gene expression of GAPDH.

### 2.6. Histology

In histology analysis, the punctured intervertebral discs were harvested and fixed in 4% formaldehyde for 24 h. Then, the tissue was decalcified in 10% ethylenediaminetetraacetic acid for 1 month and embedded with routine paraffin and cut into 5 *μ*m sections. To observe the pathological change of the tissue, hematoxylin-eosin (H&E) staining and safranin O/fast green staining was investigated according to protocols. The histological images were captured with a microscope (Axio, Carl Zeiss, Oberkochen, Germany). Subsequently, the modified Thompson grade scale was used to evaluate the severity of intervertebral disc degeneration. The histological results were investigated by two independent authors.

### 2.7. Immunohistochemistry (IHC)

Besides H&E and safranin O/fast green staining, the sectioned tissue was stained for expression of NGF. The NGF primary antibody (cat. no. AF-556-NA; R&D Systems) was used for incubation at 4°C overnight, and the next process was conducted using IHC kit (cat. no. K5007; Agilent DAKO Inc., CA, USA). The nuclei of cells were counterstained using hemalum (cat. G1004; Servicebio Inc., China). After photographed with a microscope (Axio, Carl Zeiss, Oberkochen, Germany), five sections were randomly selected in each sample for analysis of positive cells and compared among each groups.

### 2.8. Statistical Analysis

The data are recorded using mean ± SD and calculated with GraphPad Prism software (version 6; GraphPad Software Inc.). In multiple group analysis, one-way or two-way ANOVA with post hoc Tukey's multiple comparison test was used. For two group comparisons, the two-sided Student's *t*-test was used. *p* value < 0.05 was set as statistical significance.

## 3. Result

### 3.1. HQGZ Formula Attenuated Discogenic LBP in Rats

**Figure 1:**
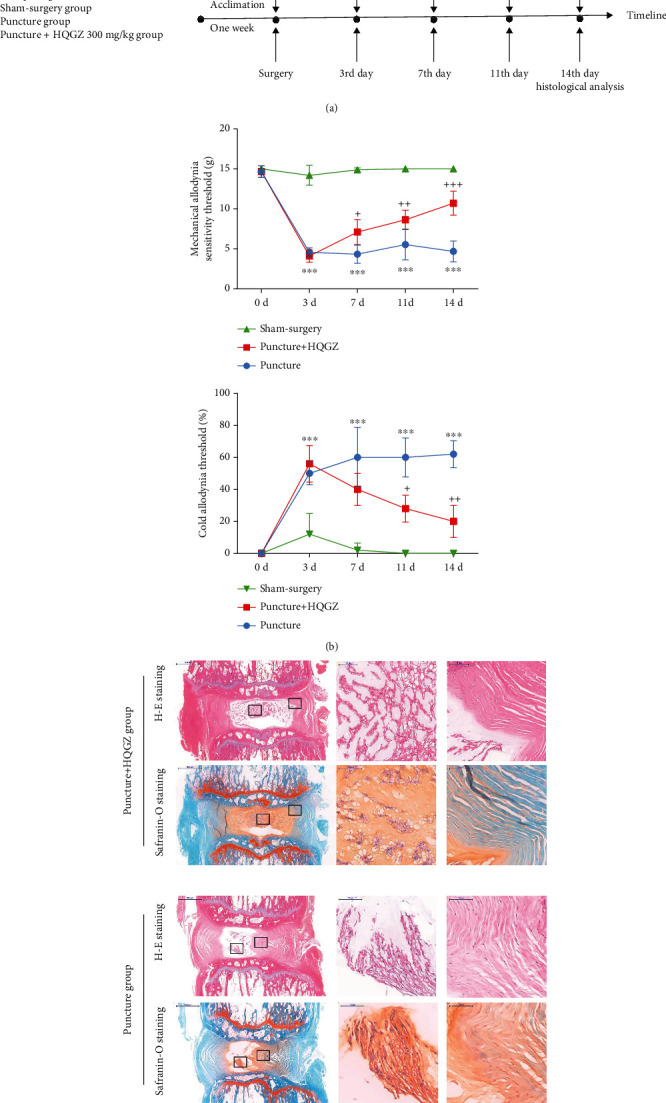
HQGZ formula significantly attenuated discogenic LBP in rats. (a) An illustration of the timeline for model establishment, drug delivery, and behavioral analysis. (b) Intragastric administration of HQGZ formula at a dose of 300 mg/kg/day for 2 weeks resulted in significantly attenuated mechanical and cold allodynia in the puncture+HQGZ group compared to the puncture group on days 3–14. (c) Histological analysis suggested that HQGZ significantly ameliorated disc degeneration, which is the primary mechanism involved in the alleviation of discogenic LBP. (^∗∗∗^ <0.001 when compared between the sham-surgery and puncture groups. +<0.05, ++<0.01, and +++<0.001 when compared between the sham-surgery and puncture+HQGZ groups. *n* = 5 in each animal group. The modified Thompson grade scale was grade 4 and grade 2 for the puncture group and puncture+HQGZ group. The data are shown as the mean ± SD. Two-way ANOVA with Tukey's multiple comparison test was used for multiple group comparisons.).

The animal showed significant discogenic LBP after puncture of the lumbar IVDs with an 18-gauge needle, as evidenced by a decrease of mechanical hyperreflexia threshold and increase of cold hyperreflexia threshold compared to the sham-surgery group from the days 3–14, as depicted in Figures 1(a) and 1(b). However, HQGZ intragastrically given with a dosage of 300 mg/kg/day significantly reversed the trend, as evidenced by a gradual increase of mechanical hyperreflexia threshold and decrease of cold hyperreflexia threshold when made comparison with puncture group on days 3–7.

Furthermore, we performed a histological analysis to evaluate the therapeutic effect of HQGZ for disc degeneration. As shown in Figure 1(c), the rats treated with HQGZ had slight intervertebral disc degeneration, as evidenced by slight proliferation of chondrocytes inside the nucleus pulposus (NP) and slightly disorganized annulus fibrosus (AF), while the rats from the puncture group had moderate degeneration. Therefore, HQGZ significantly ameliorated LBP and disc degeneration.

### 3.2. Wogonin Is the Primary Bioactive Factor in HQGZ according to Network Pharmacological Analysis

**Figure 2:**
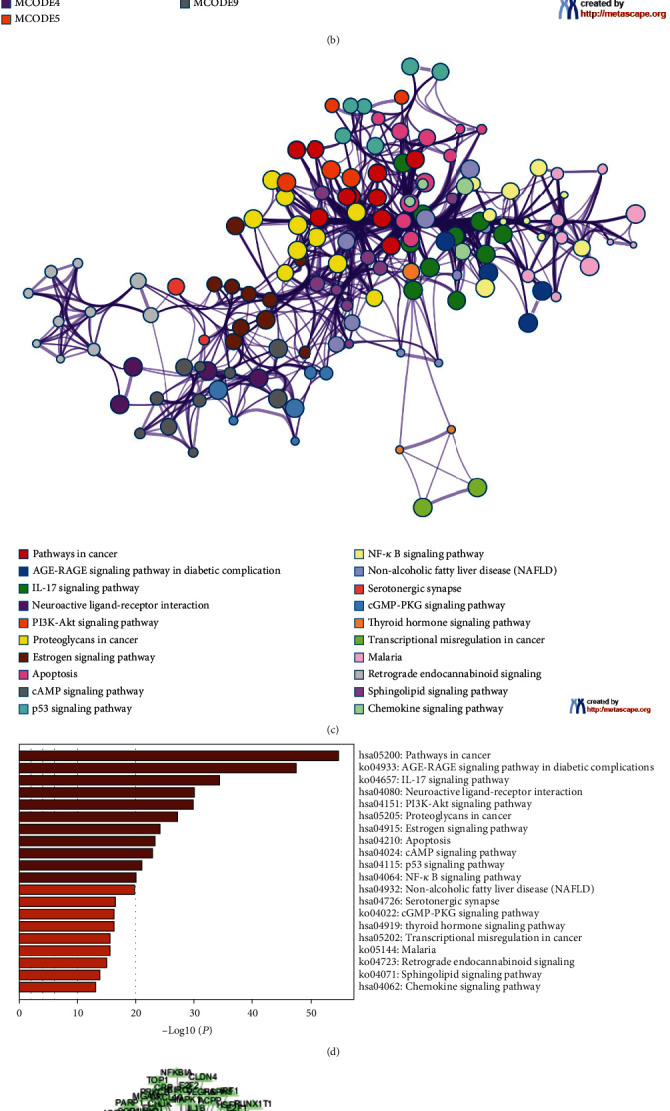
Network pharmacology analysis of HQGZ formula in discogenic LBP. (a) There were 257 potential therapeutic targets in the HQGZ formula for discogenic LBP. (b) A total of 9 MCODE networks were constructed using PPI enrichment analysis of the 257 therapeutic targets. Different colors represent different MCODE networks. (c) GO enrichment analysis of 257 therapeutic targets (colored according to *p* values). (d) KEGG enrichment analysis of 257 therapeutic targets (colored according to *p* values). (e) Active ingredients and target networks of the HQGZ formula. Red ovals represent active ingredients and green triangles represent targets. (f) The analysis of potential therapeutic candidates (i.e., wogonin, quercetin, and kaempferol) assumed to be the most likely bioactive chemical components of the HQGZ formula for discogenic LBP.

To identify which factor was the primary bioactive ingredients from HQGZ, we conducted a network pharmacology analysis (Figure 2). Based on TCMSP database, 243 active ingredients of HQGZ and 276 targets of bioactive components were obtained. We removed duplicate data and genes that could not be queried in the UniProt database. Then, 7,748 LBP genes were collected based on GeneCards, DisGeNET, OMIM, and PharmGKB databases. Then, a Venn diagram of the 276 composite ingredients and 7,748 LBP targets was drawn (Figure 2(a)), resulting in a total of 257 overlapping targets, which were considered as the core genes of HQGZ in LBP treatment.

To fully elucidate the underlying mechanism of HQGZ in the treatment of LBP, 257 core genes were subjected to PPI analysis. The molecular complex detection (MCODE) algorithm was used for demonstration of densely connected network ingredients. Pathway and process enrichment analyses were independently applied to each MCODE component, and the best scoring terms of the three *p* values were retained as the functional description of the corresponding components (Supplementary Table 1). As shown in Figure 2(b), a total of nine MCODEs were identified from the constructed network, involving pathways related to cancer, human T-cell leukemia virus type 1 infection, human cytomegalovirus infection, PI3K-Akt signaling, and neuroactive ligand-receptor interactions.

Next, GO and KEGG enrichment analyses were performed. GO enrichment analysis showed that the main enriched terms of the target genes included responses to hormones, inorganic substance, lipid, xenobiotic stimulus, and oxygen levels, as well as positive regulation of protein phosphorylation, positive regulation of cell death, regulation of apoptosis signaling pathway, and neurotransmitter receptor activity (Supplementary Table 2 and Figure 2(c)). Furthermore, KEGG analysis revealed that HQGZ could affect multiple pathways, including pathways related to cancer, lipid and atherosclerosis, chemical carcinogenesis-receptor activation, IL-17 signaling pathway, PI3K-Akt signaling pathway, chemical carcinogenesis-reactive oxygen species, MAPK signaling pathway, neuroactive ligand-receptor interaction, alcoholic liver disease, and pathways of neurodegeneration-multiple diseases (Supplementary Table 3 and Figure 2(d)).

To further explore the factor that played a key role in LBP treatment by HQGZ from a systems and holistic perspective, we constructed a network using Cytoscape (Figure 2(e)). Network analysis showed that the top three active ingredients were quercetin, kaempferol, and wogonin. The active ingredient-target network diagram was constructed for each ingredient (Figure 2(f)). These three compounds acted on multiple LBP targets and were considered to play a key role in LBP treatment with HQGZ.

### 3.3. Treatment with Wogonin Ameliorated the LBP and Disc Degeneration

**Figure 3:**
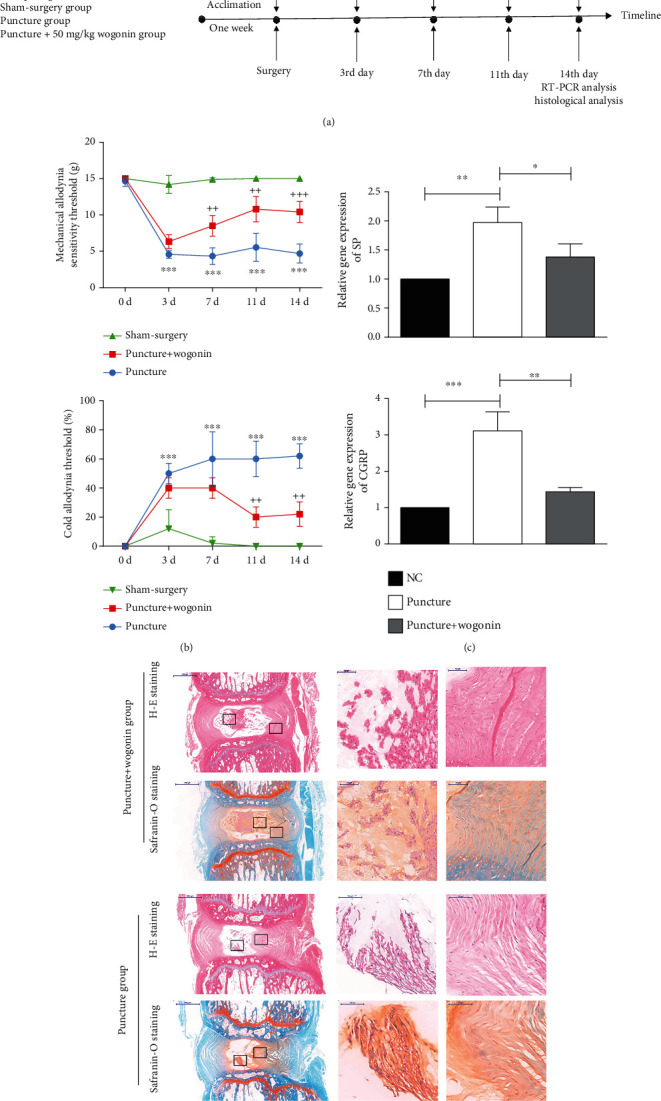
Wogonin had significant therapeutic effects for LBP and IDD. (a) An illustration of the timeline for wogonin administration and behavioral analysis. (b) The behavioral analysis showed that treatment with wogonin at a dose of 50 mg/kg/day via an intraperitoneal injection significantly increased the threshold of mechanical hyperreflexia and decrease the threshold of cold hyperreflexia compared to the puncture group. (c) RT-PCR analysis of SP and CGRP in the DRGs verified the antianalgesic effect of wogonin in rats, as evidenced by the down- and upregulation of both in the wogonin+puncture group. (d) Treatment with wogonin attenuated puncture-induced IDD. (^∗∗∗^ <0.001 when compared between the sham-surgery and puncture groups. ++<0.01 and +++<0.001 when compared between the sham-surgery and puncture+wogonin groups. *n* = 5 in each animal group. The modified Thompson grade scale was grade 4 and grade 3 for the puncture group and puncture+wogonin group. The data are shown as the mean ± SD. One-way or two-way ANOVA with Tukey's multiple comparison test was used for multiple group comparisons.).

We used network pharmacology analysis to determine the therapeutic effect of wogonin. As shown in Figures 3(a) and 3(b), wogonin at a dose of 50 mg/kg/day significantly attenuated the puncture-induced discogenic LBP in rats, as evidenced by increased mechanical allodynia and decreased cold allodynia. In addition, we calculated the genetic expression of SP and CGRP, in the DRGs. On day 14, the animals given with wogonin with a dosage of 50 mg/kg/day had downregulation of SP and CGRP compared to the puncture group (Figure 3(c)). In addition, histological analysis revealed that wogonin had significant therapeutic effects on puncture-induced disc degeneration, suggesting a lower histological score than that of the puncture group (Figure 3(d)). Therefore, we concluded that wogonin, one of the critical components of HQGZ, had significant therapeutic effects on LBP.

### 3.4. Wogonin Suppressed NGF Upregulation to Treat LBP

**Figure 4:**
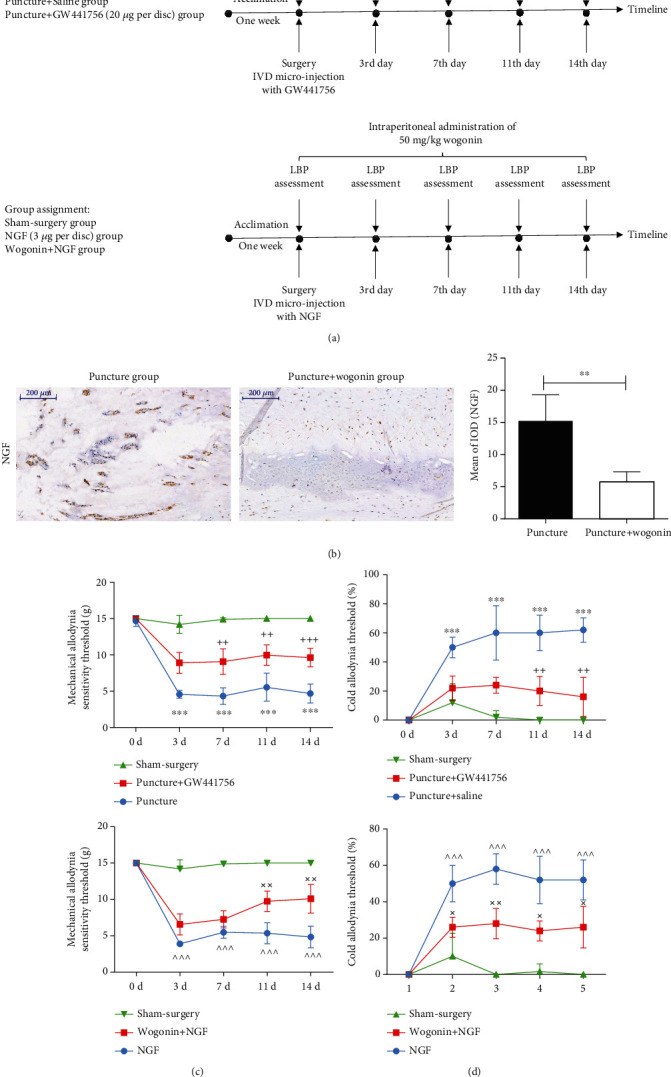
Wogonin alleviated discogenic LBP via suppressing NGF expression in IVDs. (a) An illustration of the timeline for wogonin administration, behavioral analysis, and IHC. (b) The IHC results suggested that wogonin treatment suppressed NGF expression in degenerated IVDs with discogenic LBP. (c) To confirm the role of NGF in LBP, GW441756, an inhibitor of TrkA that blocks the bioeffects of NGF, was injected, which resulted in significant amelioration of LBP in rats compared to the puncture group. (d) When NGF was injected into IVDs of rats, wogonin treatment significantly attenuated NGF-induced LBP in rats. (^∗∗∗^ <0.001 when compared between the sham-surgery group and puncture group. ++<0.01 and +++<0.001 when compared between the puncture+GW441756 group and puncture group. ^^^<0.001 when compared between the NGF group and sham-surgery group. ××<0.01 when compared between the NGF group and wogonin+NGF group. *n* = 5 in each animal group. The data are shown as the mean ± SD. Two-way ANOVA with Tukey's multiple comparison test was used for multiple group comparisons. For two group comparisons, the two-sided Student's *t*-test was used.).

A previous study suggested that NGF contributed to LBP. Thus, we investigated whether it contributed to puncture-induced LBP. As shown in Figures 4(a) and 4(b), the IHC analysis showed that NGF expression was significantly increased for 14 days after lumbar IVD was punctured compared to the sham-surgery group.

To further test the proanalgesic effects of NGF, we injected GW441756, an effective inhibitor of the NGF receptor Tkr-A, to block the biological effects of NGF in IVDs.  Figure 4(c) showed that puncture-induced discogenic LBP was significantly reversed by the use of GW441756. In addition, we injected NGF at a dose of 3 *μ*g per disc and found significantly increased mechanical and cold allodynia; however, the administration of wogonin significantly attenuated NGF-induced mechanical and cold pain (Figure 4(d)). Combined with the data that wogonin suppressed puncture-induced NGF upregulation, we concluded that wogonin ameliorated LBP via suppressing NGF overexpression in intervertebral discs.

## 4. Discussion

Here, it was suggested that HQGZ formula had significantly therapeutic effects for LBP. The network pharmacological analysis revealed that wogonin may be one of the primary bioactive ingredients. We also found that the administration of wogonin significantly ameliorated LBP based on the behavioral test and *in vivo* biochemical analysis. Finally, we revealed that the pharmacological effect of wogonin on attenuation of LBP occurred through suppressing of NGF expression. Thus, we concluded that wogonin, which is derived from the HQGZ formula, has a promising role as an effective analgesic drug for LBP treatment.

There were several theories to explain the potential therapeutic mechanism of TCM; however, bioactive ingredients were thought to be the best candidate. Bioactive ingredients play a variety of pathophysiological and cellular biological effects and thus had tremendous therapeutic effects. Here, based on the network pharmacological analysis, we confirmed that wogonin had significant therapeutic effects for IDD and discogenic LBP. Our analysis suggested that wogonin, quercetin, and kaempferol were the most likely candidates involved in the therapeutic effects of HQGZ on LBP. We selected wogonin for further investigation because previous studies suggested that wogonin had significant analgesic effects for various pain types. For example, Smith et al. revealed that topical application of wogonin significantly alleviated osteoarthritis-related knee pain [15]. In addition, Chen et al. demonstrated that application of wogonin inhibited lipopolysaccharide-induced inflammation of DRGs neurons in rat by inhibition of the TLR4-MyD88-TAK1-mediated NF-*κ*B and MAPK signaling pathways [8]. Here, it was suggested that wogonin alleviated discogenic LBP via suppressing the critical pain-related factor NGF, suggesting that the compound is a promising candidate for analgesic use.

In addition to the analgesic effect, a previous study suggested that wogonin also had various bioactivities, such as inhibition of viral replication, inhibition of inflammatory pathology, and anxiolytic and anticonvulsant effects [7]. Fang et al. suggested that wogonin attenuated the degeneration of NP via activation of the Nrf2/HO-1-SOD2-NQO1-GCLC signaling axis [16]. Moreover, Khan et al. demonstrated that wogonin was able to protect chondrocytes through the amelioration of oxidative stress, inflammation, and matrix degradation at the molecular level [5]. Furthermore, Park et al. reported that wogonin would target the MMP-3 on articular chondrocytes to play a protective role [17]. Here, it was suggested that wogonin would regulate NGF signaling pathway and anti-inflammation effect may be the primary mechanism underlying its therapeutic effects.

As an important neuropeptide, NGF is strongly correlated with discogenic LBP. In our previous study, we found that *Propionibacterium acnes*-infected IVD produced large quantities of NGF and the increased NGF was the primary factor involved in LBP [10]. Recently, specific antibodies directed against NGF were used for therapy of LBP [18]. Multiple mechanisms contribute to NGF-induced LBP. First, NGF strongly induces ingrowth of nerve fibers into the IVD, as evidenced by the previous findings that most of neurons in an inflammatory disc mode were NGF-dependent and by the study from Freemont et al. that the innervation of discs had relationship with NGF released by the microvascular blood vessels [19, 20]. Second, NGF may have biological effects that stimulate the production and release of other pain-related factors or peptides [21]. Therefore, the management of NGF expression is vital to alleviate LBP.

There are some limitations to this study. First, the pharmacological mechanism underlying NGF suppression by wogonin needs to be verified in *in vivo* studies. Second, the network pharmacology analysis revealed three bioactive components involved in LBP and the therapeutic effects of the other two ingredients (quercetin and kaempferol) were not investigated; these should be investigated in future studies. Finally, a detailed pharmacokinetics analysis of the HQGZ formula was not performed and should be performed in future studies.

## 5. Conclusions

HQGZ, a TCM herbal formula, had significant therapeutic effects on LBP. In addition, wogonin, the key bioactive ingredient of HQGZ according to the network pharmacology analysis, ameliorated LBP by suppressing the overexpressed NGF in degenerated IVDs. In the future, wogonin may be a promising drug for the alternative treatment of LBP.

## Data Availability

The datasets used and analyzed during the current study are available from the corresponding author on reasonable request.

## Supplementary Materials

Supplementary Materials Supplementary Table 1: the molecular complex detection (MCODE) algorithm analysis for functional description of the corresponding components. Supplementary Table 2: the Gene Ontology (GO) enrichment analysis for network of molecular mechanism. Supplementary Table 3: the Kyoto Encyclopedia of Genes and Genomes (KEGG) enrichment analysis for network of molecular mechanism.
