# Supplementary material for: Wogonin, a Bioactive Ingredient from Huangqi Guizhi Formula, Alleviates Discogenic Low Back Pain via Suppressing the Overexpressed NGF in Intervertebral Discs
Source: Mediators Inflamm. 2023 Feb 20;2023:4436587. doi: 10.1155/2023/4436587 (PMC9970730; doi:10.1155/2023/4436587)
Supplement: Supplementary Materials — Supplementary Table 1: the molecular complex detection (MCODE) algorithm analysis for functional description of the corresponding components. Supplementary Table 2: the Gene Ontology (GO) enrichment analysis for network of molecular mechanism. Supplementary Table 3: the Kyoto Encyclopedia of Genes and Genomes (KEGG) enrichment analysis for network of molecular mechanism. [file 4436587.f1.docx]

**Supplementary Table 1 The Molecular Complex Detection (MCODE) algorithm analysis for functional description of the corresponding components**

| MCODE | GO | Description | Log10(P) |
| --- | --- | --- | --- |
| MCODE_1 | hsa05200 | Pathways in cancer | -26.9 |
| MCODE_1 | hsa05166 | Human T-cell leukemia virus 1 infection | -23 |
| MCODE_1 | hsa05163 | Human cytomegalovirus infection | -22.9 |
| MCODE_2 | hsa05200 | Pathways in cancer | -21.3 |
| MCODE_2 | hsa04151 | PI3K-Akt signaling pathway | -19 |
| MCODE_2 | hsa04931 | Insulin resistance | -18.1 |
| MCODE_3 | hsa05200 | Pathways in cancer | -20.4 |
| MCODE_3 | hsa05161 | Hepatitis B | -18.8 |
| MCODE_3 | hsa04933 | AGE-RAGE signaling pathway in diabetic complications | -18.7 |
| MCODE_4 | hsa05417 | Lipid and atherosclerosis | -11.8 |
| MCODE_4 | hsa05033 | Nicotine addiction | -10.1 |
| MCODE_4 | hsa05208 | Chemical carcinogenesis - reactive oxygen species | -9.8 |
| MCODE_5 | hsa04080 | Neuroactive ligand-receptor interaction | -18.1 |
| MCODE_5 | hsa04061 | Viral protein interaction with cytokine and cytokine receptor | -6.8 |
| MCODE_5 | hsa04022 | cGMP-PKG signaling pathway | -5.9 |
| MCODE_6 | hsa00350 | Tyrosine metabolism | -14.7 |
| MCODE_6 | hsa00982 | Drug metabolism - cytochrome P450 | -13.2 |
| MCODE_6 | hsa00360 | Phenylalanine metabolism | -8.9 |
| MCODE_8 | hsa04024 | cAMP signaling pathway | -8.6 |
| MCODE_8 | hsa04080 | Neuroactive ligand-receptor interaction | -7.7 |
| MCODE_8 | hsa04020 | Calcium signaling pathway | -5.7 |
| MCODE_9 | hsa05417 | Lipid and atherosclerosis | -6.4 |

**Supplementary Table 2** **The Gene Ontology (GO) enrichment analysis for network of molecular mechanism**

| **GO** | **Category** | **Description** | **Count** | **%** | **Log10(P)** | | **Log10(q)** |
| --- | --- | --- | --- | --- | --- | --- | --- |
| GO:0009725 | GO Biological Processes | response to hormone | 74 | 28.79 | | -56.26 | -51.91 |
| GO:0010035 | GO Biological Processes | response to inorganic substance | 64 | 24.9 | | -54.43 | -50.38 |
| GO:0071407 | GO Biological Processes | cellular response to organic cyclic compound | 62 | 24.12 | | -52.63 | -48.76 |
| GO:0009410 | GO Biological Processes | response to xenobiotic stimulus | 55 | 21.4 | | -49.15 | -45.51 |
| GO:0071396 | GO Biological Processes | cellular response to lipid | 55 | 21.4 | | -43.68 | -40.18 |
| GO:0070482 | GO Biological Processes | response to oxygen levels | 42 | 16.34 | | -36.7 | -33.31 |
| GO:0051272 | GO Biological Processes | positive regulation of cellular component movement | 52 | 20.23 | | -36.18 | -32.89 |
| GO:0009991 | GO Biological Processes | response to extracellular stimulus | 48 | 18.68 | | -36.16 | -32.89 |
| GO:0048545 | GO Biological Processes | response to steroid hormone | 39 | 15.18 | | -35.11 | -32.02 |
| GO:0097305 | GO Biological Processes | response to alcohol | 36 | 14.01 | | -33.42 | -30.43 |
| GO:0009611 | GO Biological Processes | response to wounding | 44 | 17.12 | | -33.26 | -30.29 |
| GO:0003013 | GO Biological Processes | circulatory system process | 45 | 17.51 | | -31.91 | -28.98 |
| GO:0001934 | GO Biological Processes | positive regulation of protein phosphorylation | 52 | 20.23 | | -31.53 | -28.64 |
| GO:0010942 | GO Biological Processes | positive regulation of cell death | 48 | 18.68 | | -31.45 | -28.58 |
| GO:0045121 | GO Cellular Components | membrane raft | 37 | 14.4 | | -29.74 | -26.93 |
| GO:0048732 | GO Biological Processes | gland development | 39 | 15.18 | | -28.73 | -25.97 |
| GO:0048608 | GO Biological Processes | reproductive structure development | 39 | 15.18 | | -27.89 | -25.16 |
| GO:0030594 | GO Molecular Functions | neurotransmitter receptor activity | 25 | 9.73 | | -27.88 | -25.15 |
| GO:2001233 | GO Biological Processes | regulation of apoptotic signaling pathway | 35 | 13.62 | | -25.39 | -22.8 |
| GO:0030155 | GO Biological Processes | regulation of cell adhesion | 47 | 18.29 | | -25.38 | -22.79 |

**Supplementary Table 3** **The Kyoto Encyclopedia of Genes and Genomes (KEGG) enrichment analysis for network of molecular mechanism**

| GO | Category | Description | Count | % | Log10(P) | Log10(q) |
| --- | --- | --- | --- | --- | --- | --- |
| hsa05200 | KEGG Pathway | Pathways in cancer | 77 | 29.96 | -71.9 | -69.36 |
| hsa05417 | KEGG Pathway | Lipid and atherosclerosis | 52 | 20.23 | -60.05 | -57.81 |
| hsa05207 | KEGG Pathway | Chemical carcinogenesis - receptor activation | 39 | 15.18 | -39.78 | -38.01 |
| hsa04657 | KEGG Pathway | IL-17 signaling pathway | 28 | 10.89 | -35.01 | -33.47 |
| hsa04151 | KEGG Pathway | PI3K-Akt signaling pathway | 40 | 15.56 | -32.09 | -30.6 |
| hsa05208 | KEGG Pathway | Chemical carcinogenesis - reactive oxygen species | 34 | 13.23 | -31.74 | -30.31 |
| hsa04010 | KEGG Pathway | MAPK signaling pathway | 36 | 14.01 | -30.11 | -28.81 |
| hsa04080 | KEGG Pathway | Neuroactive ligand-receptor interaction | 38 | 14.79 | -29.27 | -27.98 |
| hsa04936 | KEGG Pathway | Alcoholic liver disease | 27 | 10.51 | -27.94 | -26.73 |
| hsa05022 | KEGG Pathway | Pathways of neurodegeneration - multiple diseases | 39 | 15.18 | -25.97 | -24.86 |
| hsa04115 | KEGG Pathway | p53 signaling pathway | 20 | 7.78 | -24.27 | -23.27 |
| hsa04915 | KEGG Pathway | Estrogen signaling pathway | 24 | 9.34 | -23.89 | -22.93 |
| hsa04024 | KEGG Pathway | cAMP signaling pathway | 28 | 10.89 | -23.85 | -22.91 |
| hsa05140 | KEGG Pathway | Leishmaniasis | 19 | 7.39 | -22.11 | -21.21 |
| hsa04926 | KEGG Pathway | Relaxin signaling pathway | 21 | 8.17 | -20.32 | -19.52 |
| hsa05202 | KEGG Pathway | Transcriptional misregulation in cancer | 22 | 8.56 | -17.8 | -17.1 |
| hsa04931 | KEGG Pathway | Insulin resistance | 18 | 7 | -17.67 | -16.97 |
| hsa04726 | KEGG Pathway | Serotonergic synapse | 18 | 7 | -17.16 | -16.48 |
| hsa04022 | KEGG Pathway | cGMP-PKG signaling pathway | 20 | 7.78 | -16.65 | -15.99 |
| hsa05221 | KEGG Pathway | Acute myeloid leukemia | 14 | 5.45 | -15.32 | -14.71 |
